# Preparation and analysis of tobacco glycosides, and the relationship between glycoside aglycones and pyrolysis products: a review

**DOI:** 10.3389/fmolb.2026.1777197

**Published:** 2026-06-18

**Authors:** Kun Wang, Hongwei Zheng, Chengyu Qiu, Hongxiao Yu, Xinhua Song, Yong Yue, Zhe Zhao, Jiang Liu, Mengying Chen, Zhi Qiao, Donghai Guo, Fashi Peng, Can Lyu, Mengxue Sun, Yuru Wen, Liang Chen, Jian Liu

**Affiliations:** 1 China Tobacco Shandong Industrial Company Limited, Jinan, China; 2 Institute of Tobacco Research, Chinese Academy of Agricultural Sciences, Qingdao, China; 3 Shandong University of Science and Technology, Qingdao, China; 4 Fujian Tobacco Company Nanping Company, Nanping, China

**Keywords:** aglycone, analysis, glycosides, isolation, pyrolysis, synthesis, tobacco

## Abstract

Glycosides, characterized by high stability and the ability to release characteristic aroma components during combustion, have become an important research focus for enhancing cigarette aroma quality. However, the low extraction and synthesis efficiency of tobacco glycosides, coupled with unclear pyrolysis behavior and aroma release patterns, have hindered their application potential in cigarettes. This paper systematically reviews green extraction techniques and efficient synthesis methods for tobacco glycosides, analyzes the application of modern analytical tools such as chromatography-mass spectrometry and nuclear magnetic resonance in glycoside structural identification, and focuses on exploring the mechanisms by which aglycone types influence aroma release pathways and product compositions during pyrolysis. This research provides a theoretical foundation for the precise application of glycosides in cigarettes, holding significant practical value for advancing quality enhancement, aroma improvement, and sustainable development in the tobacco industry.

## 1 Introduction

Globally, restrictions on tar content in tobacco products are becoming increasingly stringent, making the advancement of low-tar cigarettes an inevitable direction for the tobacco industry. Since 2000, cigarette tar content in China has significantly decreased and has been effectively regulated. However, this has been accompanied by a reduction in aromatic components in smoke, resulting in insufficient cigarette flavor (Wang et al., 2023). Aroma is a core factor in evaluating tobacco leaf quality. The application of free-state aroma components is limited due to their high volatility, short retention time, and poor release uniformity. Consequently, research and development of latent aroma components are gaining widespread attention. Against this backdrop, the development of tobacco fragrance and the application of aroma compensation technologies are particularly crucial for the current research and development of low-tar cigarettes. Aroma precursor compounds undergo complex evolution throughout the entire chain from tobacco processing to combustion. Their transformation pathways and degradation products significantly influence the formation of aroma profiles and quality. These compounds primarily include carotenoids, cembrane, ladanum, nitrogen-containing compounds, and glycosides (Liang et al., 2022; Piršelová and Jakubčinová, 2025; Yue et al., 2015). Among these, glycosides exhibit excellent stability and can degrade into volatile components during cigarette smoking, enzymatic hydrolysis, acid hydrolysis, or pyrolysis (Muradova et al., 2023). They serve as important aroma precursor compounds in tobacco (Ortiz-Serrano and Gil, 2007). The primary function of glycosides lies in synergistically enhancing tobacco’s aroma quality and sensory comfort. As directly addable fragrance, they can replace volatile or unstable flavor components, optimizing the compatibility of flavor systems. This addition method not only helps avoid aroma loss caused by direct use of volatile flavor enhancers, but also better preserves the integrity of tobacco’s natural aroma.

Glycosides are vital natural phytochemicals metabolized from flavonoids, anthraquinones, terpenes, and other precursors (Chang et al., 2025). They form through dehydration condensation between the hemiacetal hydroxyl group of a monosaccharide or oligosaccharide and functional groups such as hydroxyl, amino, or sulfhydryl in an aglycone molecule. In vegetables, fruits, and tea, glycosides also serve as important aromatic precursors, capable of re-releasing flavor-related glycoside aglycones during storage, processing, and consumption (Su et al., 2025). Glycoside aroma precursor compounds primarily encompass terpene and C_13_-norisoprenoid aroma aglycones. Aglycones bind to monosaccharide/disaccharide glycosyl groups, and achieve targeted release of aroma components through glycosidic bond cleavage under thermal/enzymatic action. Glycosides contain diverse functional groups, including hydroxyl and aldehyde groups, which degrade to produce multiple flavor compounds meeting cigarette sensory requirements. Terpene glycosides serve as important aroma precursors in tobacco, with some components used as flavor enhancers. Flavonoid glycosides, abundant bioactive components, convert into flavor compounds during combustion. Glycolipids not only enhance moisture retention but also serve as important smoke aroma precursors (Wu et al., 2023).

During tobacco combustion, its internal chemical composition undergoes dynamic evolution. This transformation pathway and final smoke composition are primarily regulated by combustion temperature and oxygen supply conditions, with varying conditions yielding distinct combustion mechanisms and products (Czech et al., 2024). The introduction of pyrolysis technology has expanded research from exploring the correlation mechanisms between tobacco and smoke components to analyzing the pyrolysis behavior of aroma precursor monomers (Baker and Bishop, 2005). For an in-depth investigation into the contribution of fragrance and flavor compounds to smoke composition, pyrolysis serves as an effective screening tool to evaluate their pyrolysis behavior during cigarette smoking and systematically analyze pyrolysis products. The slow release of aglycones in tobacco enables glycoside to uniformly enhance aroma during smoking, with pyrolysis being a critical step in combustion (Li et al., 2006). Thermal analysis methods have become essential for studying the pyrolysis behavior of glycoside aroma precursors and their aroma release patterns. This approach clarifies their pyrolysis pathways and product compositions, significantly advancing the application of such compounds in high-temperature processing and cigarette aroma enhancement (Worzakowska and Ścigalski, 2014).

This paper systematically reviews green extraction techniques and efficient synthesis methods for tobacco glycosides. It analyzes the application of modern analytical tools such as chromatography-mass spectrometry and Nuclear Magnetic Resonance (NMR) in glycoside structural identification. Furthermore, it delves into the structure-activity relationships between different aglycone structures (phenol, terpene, C_13_-norisoprenoid, and other aglycones) and their pyrolysis products, while examining the influence of pyrolysis temperature on aroma release behavior.

## 2 Isolation and synthesis of glycoside compounds in tobacco

Tobacco glycosides constitute an important class of aroma precursor compounds, which are stable and odorless at ambient temperatures, they undergo pyrolysis during cigarette combustion to release volatile aromatic components. This process markedly enhances smoke quality by improving aroma richness, stability, and persistence (Liu et al., 1998). The extraction and separation of tobacco glycosides, such as flavonoid glycosides, sucrose esters, and polysaccharides, constitute important steps for their research and utilization. Based on the physicochemical properties of target compounds and research requirements, the following methods are currently predominant.

### 2.1 Separation of glycosides in tobacco

#### 2.1.1 Hot water extraction

A classic and straightforward method for extracting polysaccharide from tobacco. Chang et al. (2024) reported the purification and separation of three polysaccharide fractions using DEAE-52 cellulose chromatography columns after hot water extraction of tobacco polysaccharides. This method is typically followed by chromatographic refinement and is suitable for the preliminary extraction of polysaccharides.

#### 2.1.2 Solvent extraction

A widely applied separation method, where solvents can be selected based on target compound properties, including conventional organic/aqueous solvents and emerging green solvents. For economically valuable compounds, extraction methods should be optimized according to tobacco physicochemical characteristics. Xu et al. (2025) analyzed the tobacco metabolic composition of 168 extracts using synergistic extraction with multiple solvents. Deep Eutectic Solvents (DES) also demonstrate promising applications. Leal et al. (2023) employed natural DES to extract bioactive polyphenols and alkaloids from tobacco waste. Besides, auxiliary techniques enhancing solvent extraction efficiency include Ultrasound-Assisted Extraction (UAE) and Supercritical Fluid Extraction (SFE). UAE represents a sustainable approach to enhancing mass transfer efficiency. Yuan et al. (2018) reported a simple and efficient method based on UAE, the conditions of which were optimized by response surface methodology, for the analysis of non-volatile neutral glycosides originating from tobacco leaves. SFE, as a highly efficient and environmentally friendly technology, is particularly suited for enriching complex mixtures. It has been isolated structurally complex sucrose esters from tobacco (Zhu et al., 2023).

#### 2.1.3 Chromatographic separation techniques

Core methods for the separation and purification of glycoside compounds. The synergistic application of multiple chromatographic techniques meets diverse requirements. High Performance Gel Permeation Chromatography (HPGPC) is employed to analyze polysaccharides from discarded tobacco leaves (Wang Y. et al., 2025). High-sensitivity, high-resolution coupled chromatography techniques, such as Ultra High Performance Liquid Chromatography-Quadrupole-Time of Flight Mass Spectrometry (UPLC-Q-TOF MS) and Gas Chromatography-Mass Spectrometry (GC-MS) are utilized for qualitative and quantitative analysis of glycosides in complex samples, such as tobacco glandular trichome secretions (Liu et al., 2024). Besides, Solid-phase extraction (SPE) serves as an efficient sample preparation method for selectively isolating specific glycosides from complex matrices. He et al. (2015) based on a fast isolation of extracts obtained by HP-20 SPE and methanol elution, for estimating glycosidically bound aroma compounds in flue-cured tobacco.

#### 2.1.4 Enzyme-assisted extraction

This method utilizes enzymatic hydrolysis to enhance extraction efficiency and selectivity for target compounds, particularly suited for releasing glycoside aglycones. Miksovsky et al. (2024) developed a sequential enzymatic hydrolysis combined with a SFE process for efficient extraction of flavonoid glycoside aglycones from apple pomace, such as quercetin and kaempferol, applicable to processing glycoside compounds in tobacco.

The comparison of glycoside separation methods in tobacco is summarized in Table 1. For comprehensive glycoside analysis of tobacco samples, a combined approach utilizing solvent extraction coupled with UPLC-Q-TOF MS achieves the effective separation and identification. At the preparative level, sequential operations of solvent extraction and column chromatography remain the practical method for obtaining purified glycosides for subsequent characterization or application.

**TABLE 1:** Comparison of separation methods for glycosides in tobacco.

Method	Principle	Applicable scope
Hot water extraction	Utilizes high-temperature water to extract highly polar glycosides	Simple operation, suitable for preliminary extraction, requires further purification
Solvent extraction	Select organic or green solvents based on target compound polarity	Highly flexible, can be combined with ultrasound, supercritical fluid, etc.
Chromatographic separation	Separate target glycosides using different chromatographic columns	High resolution and sensitivity, suitable for qualitative and quantitative analysis
Enzyme-assisted extraction	Enzymatic hydrolysis of glycosidic bonds to release target compounds	Enhances selectivity, suitable for releasing aglycones

### 2.2 Synthesis of glycoside compounds in tobacco

The synthesis of tobacco glycosides primarily involves chemical and biological methods.

#### 2.2.1 Chemical synthesis methods

Chemical synthesis methods focus on glycosylation reactions between glycosyl group donors and aglycones, enhancing selectivity and yield through catalyst and reaction condition optimization. In chemical synthesis of glycosides, researchers have developed multiple efficient approaches, such as the Koenigs-Knorr method, phase transfer catalysis, the Schmidt method, the Helferich method, and the Fischer method (Zhang et al., 2014). The Koenigs-Knorr method is a classic glycosylation method. This reaction produces few byproducts and obtains high yields, but it requires the use of expensive silver salt catalysts and involves harsh reaction conditions. Using this method, geranyl-β-D-glucopyranoside, cis-3-hexenyl-β-D-glucopyranoside, menthyl-β-D-glucopyranoside, 3-oxo-α-ionol-β-D-glucoside, 5-methyl furfuryl alcohol-β-D-glucoside, and cedrol-β-D-glucoside are synthesized (Yang et al., 2007; Zhang et al., 2016). The phase transfer catalysis method offers advantage such as mild reaction conditions and simple operation. Using this method, α-ionol-β-D-glucoside, furfuryl alcohol-β-D-glucoside, homofuraneol-β-D-glucoside, maltol-β-D-glucoside, and carvacrol-β-D-glucoside are synthesized (Zhang et al., 2017; Zhang et al., 2020). The ionic liquid catalyzed method provides a route for the synthesis of glycosides. Using this method, guaiacol-β-D-glucoside, 4-methylguaiacol-β-D-glucoside, thymol-β-D-glucoside, and isoeugenol-β-D-glucoside are synthesized (Zhang et al., 2024b; Zhang et al., 2025b).

#### 2.2.2 Biosynthetic methods

Biological methods leverage the efficient biocatalytic activity of enzymes and microorganisms, offering significant advantages such as mild reaction conditions and excellent stereoselectivity. Feng et al. (2021) discovered that UGT708N1 and UGT708N2, specifically expressed in tobacco, catalyze the C-glycosylation of 2-hydroxyflavanones and 2-hydroxynaringenin C-glucoside, forming flavone mono-C-glycosides and di-C-glycosides, respectively. Huang et al. (2025) achieved *de novo* synthesis of 23 phenylethanoid glycoside derivatives in tobacco via heterologous expression of different combinations of the functional genes. Heiling et al. (2021) reported the glycosylation function of uridine diphosphate glycosyltransferases in tobacco biosynthesis of 17-hydroxygeranyllinalool diterpene glycosides. These studies provide novel insights for the large-scale production of glycoside compounds.

## 3 Analytical methods for glycosides in tobacco

As important secondary metabolites, glycosides in tobacco encompass diverse types, including flavonoid glycosides, diterpene glycosides, phenolic acid glycosides, and aroma precursor glycosides, significantly influencing tobacco quality. Accurate analysis of these structurally diverse glycosides requires the integrated application of multiple modern analytical techniques (Table 2).

**TABLE 2:** Comparison of analytical methods for glycosides in tobacco.

Analytical method	Advantages	Disadvantages
HPLC	Lower cost, stable operation	Difficult to perform structural identification without mass spectrometry coupling
LC-MS/MS	No derivatization required, high sensitivity and selectivity in MRM mode	Expensive instrumentation, demanding high operational requirements
High-resolution mass spectrometry	Suitable for non-targeted screening, provides accurate molecular weight	High cost, complex data processing
GC-MS	Suitable for volatile aglycone analysis, complementary to LC-MS technology	Requires derivatization or enzymatic pretreatment
NMR	Can resolve fine structure (linkage sites, configuration, glycosidic bond type)	Expensive instrumentation, high sample purity requirements
Spectroscopic techniques (FTIR/UV)	Simple operation, low cost, provides functional group information or preliminary identification	Only assists in identification, lacks specificity and is susceptible to interference
Enzymatic hydrolysis and derivatization	High enzymatic hydrolysis specificity, derivatization enhances detection sensitivity	Enzymatic hydrolysis is time-consuming, derivatization steps are cumbersome

### 3.1 Chromatography-mass spectrometry

With its high-efficiency separation and precise identification capabilities, chromatography-mass spectrometry has become an indispensable core analytical tool in tobacco glycoside research.

HPLC, often coupled with Ultraviolet (UV) or Fluorescence Detectors (FLD), is suitable for the separation and quantitative analysis of glycosides. This method combines high separation efficiency, good method stability, and cost-effectiveness. Keinänen et al. (2001) reported a HPLC coupled Ultraviolet-Visible (UV-vis) screening procedure of jasmonate-induced increases in diterpene glycosides in *Nicotiana attenuata*. However, UV/FLD detection alone is often insufficient for definitive structural identification of unknown glycosides, necessitating integration with mass spectrometry or reference standards. For example, based on High Performance Liquid Chromatograph Electrospray Ionization Linear Ion Trap Tandem Mass Spectrometry (HPLC-ESI-LIT/MS^n^) coupled with Electrospray Ionization Orbitrap Mass Spectrometry (ESI-Orbitrap-MS), eleven alcoholic glycosides, eight phenolic glycosides, two ester glycosides, and an indole glycoside were identified from tobacco (Jia et al., 2017).

Liquid chromatography-tandem mass spectrometry (LC-MS/MS) is currently the widely applied technique, particularly suited for structural identification and precise quantification of complex glycosides (Pang et al., 2007). Its advantage lies in the direct analysis of polar and thermally unstable glycosides without derivatization. Structural confirmation is achieved through characteristic fragment ions generated by MS/MS: aglycone ions, glycosidic fragment ions, and characteristic neutral losses (Wu et al., 2023). Multiple reaction monitoring (MRM) mode enables highly sensitive and selective quantification of trace target glycosides in complex matrices. Ding et al. (2015) performed a systematic screening and characterization of glycosides in tobacco leaves by liquid chromatography with atmospheric pressure chemical ionization tandem mass spectrometry. Based on neutral loss and product ion scan, 39 glycosides linked with monosaccharides, 18 glycosides linked with disaccharides, and 7 glycosides linked with trisaccharides were detected.

High-resolution mass spectrometry, particularly when coupled with UHPLC, plays an important role in non-targeted metabolomics analysis. UPLC-Q-TOF MS provides precise molecular weights and, combined with MS/MS fragmentation data, enables high-throughput screening and preliminary identification of diverse glycosides in tobacco extracts.

GC-MS is primarily used for analyzing volatile or semi-volatile glycoside derivatives, especially glycosides serving as aroma precursors such as volatile phenols and terpene glycosides. However, GC-MS analysis of glycosides typically requires prior chemical derivatization or enzymatic hydrolysis to release volatile aglycones. Cai et al. (2014) established an β-glucosidase hydrolysis method followed by GC-MS using a series optimization approach, and the proposed method was successfully carried out to analyze flue-cured tobacco, sun-cured tobacco, and oriental tobacco.

### 3.2 NMR spectroscopy

NMR spectroscopy, particularly the Heteronuclear Single Quantum Coherence (HSQC) spectrum in two-dimensional NMR, offers unparalleled advantages in resolving the fine structure of glycoside compounds. It enables precise identification of their linkage sites, glycosyl group configurations, and glycosidic bond types. Tian et al. (2023) found that 2D HSQC NMR was particularly effective for analyzing cellulose content in tobacco, which was simple, reliable, and environmentally friendly. NMR spectroscopy clearly resolves the carbon and hydrogen signals at different positions on the sugar ring, thereby determining the glycosidic bond linkage site.

### 3.3 Spectroscopic techniques

Fourier Transform Infrared Spectroscopy (FTIR) and UV-Vis Spectroscopy are commonly employed as supplementary methods. FTIR provides information on functional groups within glycoside molecules, such as glycosidic bond characteristics and characteristic absorption peaks of sugar rings (Liu and Ding, 1988). UV-Vis spectroscopy can be used for preliminary identification and quantification of glycosides possessing specific chromophores (Pang et al., 2007).

### 3.4 Enzymatic hydrolysis and derivatization methods

Enzymatic hydrolysis employs highly specific glycosidases such as β-glucosidase to selectively hydrolyze specific glycosidic bonds, releasing aglycones. The released aglycones are typically more amenable to analysis than their glycoside forms. This method is particularly suitable for the total quantification of a specific glycoside class or the indirect quantification of glycosides derived from specific aglycones. Derivatization is primarily employed to adapt glycosides or their hydrolysis products to the requirements of specific detectors. For example, flue-cured tobacco glycosides were estimated by capillary GC-MS, combining with SPE, enzymatic hydrolysis, and trifluoroacetylation derivatization, fourteen glucosides, one rutinoside, and three vicianosides were identified (He et al., 2015).

## 4 Relationship between glycoside aglycones and pyrolysis products

The pyrolysis behavior of glycosides and their aroma release are jointly regulated by glycosyl composition and linkage type, aglycone structure, and pyrolysis temperature (Table 3). The glycosyl structure influences the thermal stability, dissociation pattern, and byproduct formation of glycosides. The aglycone determines the fundamental aroma characteristics of pyrolysis products. Pyrolysis temperature regulates the extent and pathway of chemical bond cleavage, thereby affecting the release efficiency and composition of products.

**TABLE 3:** Relationship between glycoside structures and pyrolysis products.

Glycosyl structure	Aglycones structure	Pyrolysis temperature	Pyrolysis products	Aroma release
Glucose monosaccharide (β-O-glycosidic bond)	Phenols	300 °C–900 °C	Primarily as aglycones, with a few phenols	Spicy, woody
	Terpenes	300 °C–900 °C	Primarily as aglycones, with a few terpenes	Floral, fruity
	C_13_- norisoprenoid	300 °C–900 °C	Primarily ketones, with a few aglycones	Sweet, floral
	Furans	300 °C–900 °C	Primarily furan derivatives, with a few aglycones	Coffee, caramel sweet

### 4.1 Phenol aglycones of glycoside

Phenol aglycones generally contain benzene rings and hydroxyl substituents. These shared structural characteristics dictate that their pyrolysis primarily releases corresponding phenolic compounds. The glycosyl group may connects to the aglycone via a β-O-glycosidic bond, whose pyrolysis temperature directly affects aglycone release efficiency. The pyrolysis temperature determines the complexity and aroma characteristics of the final products, which is achieved by regulating the competition between glycosidic bond cleavage and secondary cleavage of the aglycones. Temperatures between 300 °C–600 °C favor the selective cleavage of glycosidic bonds and release pure aromas. Higher temperatures (900 °C) induce cleavage of the aglycone side chain or aromatic ring, increasing product and aroma complexity but potentially introducing off-aromas.

#### 4.1.1 Guaiacol

Guaiacol possesses both smoky and fragrant aromas. Zhang et al. (2025a) added synthetically guaiacol-β-D-glucoside to cigarettes and subjected them to pyrolysis at 300, 600, and 900 °C, respectively. The results showed that guaiacol remained the pyrolysis product (Table 4). However, guaiacol was the sole product at 300 °C, while the number of products significantly increased at 900 °C, including catechol and 2-hydroxybenzaldehyde in addition to guaiacol.

**TABLE 4:** Representative glucosides and their pyrolysis products.

Aglycone structure	Aglycone name	Pyrolysis temperature	Major pyrolysis products	References
Phenols	Guaiacol	300 °C–900 °C	Primarily guaiacol, and small amounts of catechol and phenol at 900 °C	Zhang et al. (2025a)
	4-Methylguaiacol	300 °C–900 °C	Primarily 4-methylguaiacol, and 4-methylcatechol, p-methylphenol, and 2,3-dimethylphenol increase at 900 °C	Zhang et al. (2025b)
	Carvacrol	300 °C–900 °C	Primarily carvacrol, and small amounts of 4-methyl-2-allylphenol and 4-methyl-4-penten-2-ol at 900 °C	Zhang et al. (2024a)
	Maltol	300 °C–900 °C	Primarily maltol at 600 °C–900 °C	Zhang et al. (2020)
	Thymol	300 °C–900 °C	Primarily thymol, and 2,3-dimethylphenol and 2,3-dihydro-2-methylbenzofuran increase at 900 °C	Zhang et al. (2024b)
	Vanillin	150 °C–500 °C	Primarily vanillin, and relatively low vanillin content at 150 °C	Shang et al. (2015)
	Ethyl vanillin	250 °C–900 °C	Primarily ethyl vanillin, and aromatic rings increase at 900 °C	Wang H et al. (2025)
Terpenes	Perillol	150 °C–900 °C	Primarily perillanol and small amounts of perillylacetate at 200 °C–900 °C	Wang Y et al. (2025)
	Geraniol	200 °C–700 °C	Primarily geraniol with few citronellene and β-pinene at 300 °C–500 °C	Cheng (2016)
C13- norisoprenoid	3-Oxo-α-ionol	300 °C–900 °C	Primarily megastigmatrienone and their isomers, with small amounts of 3-oxo-α-ionol at 600 °C–900 °C	Wang et al. (2012)
	7,8-Dihydro-3-oxo-α-ionol	300 °C–900 °C	Large amounts of megastigmatrienone and its isomers at 600 °C–900 °C	Wang et al. (2012)
Furans	Furfuryl alcohol	300 °C–900 °C	Furfuryl alcohol, furfural, 5-methyl-2-furaldehyde, 5-hydroxymethylfurfural, furan, 2-methylfuran, 2-vinylfuran	Zhang et al. (2024c)
	5-Methyl furfuryl alcohol	300 °C–900 °C	5-Methylfurfural, 5-methylfurfuryl alcohol, furfural, furfuryl alcohol	Zhang et al. (2019)

#### 4.1.2 4-Methylguaiacol

4-Methylguaiacol exhibits spicy and clove aromas. Zhang et al. (2025b) synthesized 4-methylguaiacol-β-D-glucoside and investigated its pyrolysis behavior at different temperatures using Py-GC-MS. The primary pyrolysis product of this glycoside across the 300 °C–900 °C range was 4-methylguaiacol. Results indicated that pyrolysis at 300 °C yielded only 4-methylguaiacol. As the temperature increased to 600 °C, the types of pyrolysis products increased while the relative content of 4-methylguaiacol began to decrease. At 900 °C, the product composition became more complex, including lots of components besides 4-methylguaiacol.

#### 4.1.3 Carvacrol

Carvacrol possesses pungent and woody aromas. Zhang et al. (2024a) synthesized carvacrol-β-D-glucoside. Pyrolysis behavior at 300 °C–900 °C showed that carveol remained the primary product. As the temperature increased, the type of pyrolysis products increased while the proportion of carveol correspondingly decreased. At 900 °C, products included 4-methyl-4-penten-2-ol and toluene.

#### 4.1.4 Maltol

Heat-induced cleavage of the maltol-β-D-glucoside bond released characteristic volatile components such as maltol (Zhang et al., 2020). The pyrolysis pathway of this glycoside varied significantly with temperature. At 300 °C, it exhibited low-temperature pyrolysis characteristics dominated by acetone and 1,6-anhydroglucose, without maltol formation. At elevated pyrolysis temperatures of 600 °C and 900 °C, the pathway shifted toward glycosidic bond cleavage, with maltol emerging as the primary product. Maltol glycosides released lots of aromatic compounds during pyrolysis, with maltol content peaking at 600 °C.

#### 4.1.5 Thymol

Thymol exhibits herbal and thyme aromas. The pyrolysis products of thymol-β-D-glucoside at 300 °C–900 °C were predominantly thymol, accounting for 84.93%, 77.34%, and 34.61% of the pyrolysis products at 300, 600, and 900 °C, respectively (Zhang et al., 2024b). At 300 °C, fewer pyrolysis products were produced, with product type increasing with rising temperature. At 900 °C, the highest number of products was observed, including not only thymol but also phenol, o-cresol.

#### 4.1.6 Vanillin

Vanillin exhibits sweet and vanilla aromas. Shang et al. (2015) systematically investigated the thermal weight loss characteristics and pyrolysis product composition of vanillin-β-D-glucoside across typical heating temperature ranges of non-combustible cigarettes, using Py-GC-MS and thermogravimetric-differential thermal analysis (TG-DTA). Results indicated that within the 150 °C–500 °C range, the type of pyrolysis products increased with rising temperature, primarily consisting of simple phenols and aldehydes, with vanillin exhibiting the highest relative content. Within the 200 °C–300 °C range, this glycoside achieved complete weight loss and efficiently released vanillin, while producing only minimal harmful pyrolysis products.

#### 4.1.7 Ethyl vanillin

Ethyl vanillin exhibits sweet and buttery aromas. Kang (2019) synthesized ethyl vanillin-β-D-glucoside and analyzed its pyrolysis behavior between 250 °C and 900 °C using Py-GC-MS. Results indicated that ethyl vanillin was the primary pyrolysis product, with its release decreasing as temperature increased. However, the total amount of pyrolysis products significantly increased with rising temperature. When the temperature rose from 250 °C to 900 °C, the quantities of other aromatic ring compounds besides ethyl vanillin obviously increased, as did their corresponding total peak area ratio. As the temperature increased, the ethyl vanillin content decreased from 96% at 250 °C to 63% at 900 °C. Since other aromatic ring flavor components primarily originate from the pyrolysis of ethyl vanillin, the inverse relationship between their respective contents reflects the progressively greater degradation of ethyl vanillin at elevated temperatures.

### 4.2 Terpene aglycones of glycoside

The pyrolysis products of terpene glycosides primarily involve the aglycone. Terpene aglycone possesses an unsaturated olefin double bond or hydroxyl groups. Besides glycosidic bond cleavage, releasing the aglycone during pyrolysis, the double bond may undergo isomerization, cyclization, or oxidation, producing diverse terpene derivatives that enrich aroma complexity. The release efficiency is jointly determined by the breakage temperature of glycosidic bonds and the thermal stability of aglycone. Excessively high temperatures may cause aglycone degradation or an increase in byproducts.

#### 4.2.1 Perillol

Perillol possesses anise and pungent aromas. Wang H. et al. (2025) synthesized perillol-β-D-glucoside and investigated its pyrolysis behavior using TG-DTG and Py-GC-MS. Results indicated that temperature significantly influenced product composition and relative abundance. At pyrolysis temperatures above 300 °C, the glycosidic bond was broken, yielding perillanol and other volatile components. The type of pyrolysis products markedly increased with rising temperature. Within the 300 °C–900 °C range, the pyrolysis products of perillol-β-D-glucoside primarily included compounds such as perillol and perillyl acetate. During heating, this glycoside efficiently released perillol and produced many characteristic aroma components. These components originated from glycosidic bond cleavage and subsequent pyrolysis of the glycosyl group with perillol, with perillol exhibiting the highest yield from glycosidic bond breakage.

#### 4.2.2 Geraniol

Geraniol possesses sweet and floral aromas. Cheng (2016) investigated the pyrolysis behavior of geraniol glycosides in the 200 °C–700 °C using Py-GC-MS. Results showed that geraniol release was highest between 200 °C–500 °C, peaking around 300 °C, indicating this temperature as the optimal pyrolysis condition for the glycoside. Additionally, geraniol glycoside released many aromatic components, including furfurol, citronellene, and β-pinene. These components synergistically enhanced the richness and complexity of the aroma. Pyrolysis temperature significantly influenced aroma release efficiency and product distribution. Excessively low temperatures slowed reactions and resulted in incomplete aroma release, while excessively high temperatures triggered secondary pyrolysis, increasing byproduct formation.

#### 4.2.3 Nerol

Nerol possesses sweet and orange blossom aromas. Lei et al. (2017) employed TG-DTA and differential scanning calorimetry (DSC) to investigate the pyrolysis process and kinetics of nerol-β-D-glucopyranoside. TG-DTG curves revealed a single-step degradation process, with the maximum degradation temperature varying across different heating rates (5, 10, 15 °C/min). DSC analysis indicated a primary degradation temperature range of 230 °C–310 °C, consistent with TG-DTG results, reflecting simultaneous melting and decomposition during degradation.

### 4.3 C_13_-norisoprenoid aglycones of glycoside

Many C_13_-norisoprenoid aglycones possess cyclohexene structures and serve as crucial aroma precursors in tobacco. Glycoside pyrolysis produces high-aroma compounds such as megastigmatrienone, β-damascenone, and β-ionone. Pyrolysis also involves isomerization reactions of the aglycones, yielding diverse ketone derivatives that significantly contribute to tobacco’s sweet and floral aromas.

#### 4.3.1 3-Oxo-α-ionol

3-Oxo-α-ionol exhibits a fragrance aroma. Wang et al. (2012) isolated 3-oxo-α-ionol-β-D-glucopyranoside from flue-cured tobacco using a combination of traditional column chromatography and HPLC. Under a helium atmosphere, online pyrolysis analysis of the glycoside at 300, 600, and 900 °C revealed that primary pyrolysis products, including megastigmatrienone and its isomers, 3-oxo-α-ionol, etc., 600 °C is determined as the optimal pyrolysis temperature for this glycoside.

#### 4.3.2 7,8-Dihydro-3-oxo-α-ionol

The pyrolysis behavior of 7,8-dihydro-3-oxo-α-ionol-β-D-glucopyranoside was investigated at pyrolysis temperatures (300, 600, 900 °C) under a helium atmosphere (Wang et al., 2012). Results indicated that only isophorone was produced at 300 °C, while at 600 °C and 900 °C, megastigmatrienone and its isomers were produced. At 900 °C, the yield of 3-oxo-7,8-dihydro-α-ionol significantly increased.

### 4.4 Other aglycone of glycosides

Furan aglycones possess a five-membered oxygen heterocycle structure. The heterocycle may undergo ring-opening or rearrangement during pyrolysis, producing derivatives like furan and pyran that contribute coffee and caramel-sweet aromas.

#### 4.4.1 Furfuryl alcohol

Furfuryl alcohol exhibits an alcoholic and sweet aroma. Zhang et al. (2024c) synthesized furfuryl alcohol-β-D-glucoside and analyzed its pyrolysis products. Results indicated that the primary pyrolysis product of furfuryl alcohol-β-D-glucoside between 300 °C–900 °C was furfuryl alcohol. The pyrolysis temperature significantly influenced product composition. Fewer products were produced at 300 °C, while 900 °C yielded markedly more complex mixtures. High temperature pyrolysis products included furans, 2-methylfuran, and other furan derivatives in addition to furfuryl alcohol. During cigarette smoking, this glycoside releases volatile furfuryl alcohol through glycosidic bond cleavage, with a transfer rate of 0.68% into the particulate phase of mainstream smoke.

#### 4.4.2 5-Methyl furfuryl alcohol

5-Methyl furfuryl alcohol exhibits sweet and caramel aromas. Zhang et al. (2019) synthesized 5-methyl furfuryl alcohol-β-D-glucoside. Analysis of its pyrolysis behavior revealed that, despite pyrolysis at different temperatures, the primary aroma components remained consistent: 5-methyl furfural and 5-methyl-2-furanmethanol. At temperatures of 300, 600, and 900 °C, the pyrolysis products primarily comprised furan compounds. Furan compounds generally exhibit typical aromas of coffee, grains, and fruits. The small amount of pyran compounds produced during pyrolysis exhibited a rich caramelized sweet aroma.

#### 4.4.3 Homofuraneol

Homofuraneol exhibits sweet and caramel-like aromas. Zhang et al. (2023) added homofuraneol-β-D-glucoside to cigarettes for pyrolysis and smoking. GC-MS analysis of mainstream smoke particulate matter components revealed a significant increase in homofuraneol content, with no obvious changes in other components. This confirmed that homofuraneol-β-D-glucoside pyrolysis at smoking temperatures to produce homofuraneol, with a transfer rate of 3.11% into the mainstream smoke particulate phase.

The pyrolysis behavior of glycosides with different aglycones indicates that glycosides can release corresponding aglycones through glycosidic bond cleavage, with the cleavage temperature occurring at or below 300 °C. Pyrolysis temperature generally regulates product complexity. At lower temperatures, release is primarily of a single aglycone. As temperature increases, aglycone isomerization or rearrangement reactions accompany the process, and at higher temperatures, deep cleavage of aglycones occurs, leading to more complex products. However, the parent structure of the aglycones significantly influences the fundamental aroma profile and secondary reaction pathways. The aromatic ring of phenol aglycone confers high thermal stability, allowing partial aglycones retention even at elevated temperatures. In contrast, terpene aglycones, due to the high reactivity of their unsaturated double bonds, undergo isomerization, cyclization, and other transformations without requiring high temperatures. C_13_-norisoprenoid aglycones can also produce characteristic aromas. The oxygen heterocycles in furan aglycones readily undergo ring-opening or rearrangement at high temperatures to produce furans or pyran derivatives, contributing a coffee or caramel sweet aroma. The influence of glycosyl type (monosaccharide/disaccharide, α/β configuration) on pyrolysis behavior primarily manifests in differences in glycoside pyrolysis products and glycosidic bond cleavage rates. However, current research predominantly focuses on glucosides and β-configuration glycosidic bonds, with insufficient comparative studies on other glycosyl structures.

## 5 Outlook

### 5.1 Molecular structural basis for differences in glycoside thermal stability and release behavior

Glycosides with different aglycone structures exhibit distinct optimal aroma release temperatures and thermal stability. How do structural differences in aglycones quantitatively influence the activation energy for the breakage of glycosidic bonds? In the future, a series of glycosides with gradients in electronic effects and steric hindrance could be designed. Using standardized Py-GC-MS procedures, their pyrolysis onset and maximum release-rate temperatures could be evaluated. Combined with theoretical calculations to determine the activation energy for glycosidic bond breakage, a free energy relationship model capable of predicting their pyrolysis temperature window could be constructed. This would provide a theoretical tool for the rational molecular design of heat-stable or rapid-release glycosides.

### 5.2 Establishing a quantitative structure-property relationship (QSPR) model linking glycoside structure, pyrolysis products, and sensory characteristics

The aglycone structure determines the basic aromatic profile of pyrolysis products. However, most literature is limited to qualitative descriptions of product types, lacking quantitative correlations from “glycoside structure” to “product profiles” and then to “olfactory perception,” and there is a lack of QSPR models for glycosides. Subsequent studies can utilize data on the pyrolysis products from glycoside aglycones as a training set. By extracting topological, electronic, and stereochemical descriptors of the aglycone molecules and combining them with aroma-contributing and potentially harmful compounds in the pyrolysis products, a model is established to predict the product distribution of glycosides with different structures at various temperatures. The model’s outputs can then be correlated with the sensory panel’s quantitative ratings of the products. This model enables the simulation and evaluation of a new glycoside’s aroma release potential and potential risks prior to synthesis.

### 5.3 Factors affecting pyrolysis kinetics and transfer rates in mainstream smoke

The Py-GC-MS environment of single-component differs significantly from that of the actual cigarette combustion, and literature data indicate marked differences in transfer rates of various glycoside aglycones in smoke. What factors influence the transfer rate of aroma compounds released by the pyrolysis of glycosides from the pyrolysis zone into mainstream smoke? This may be related to the volatility of the aglycone itself and its interaction with the tobacco matrix. In subsequent studies, the series of glycosides with different transfer rates can be used as probe molecules. By analyzing their volatility and their interaction characteristics with tobacco matrix components such as cellulose and lignin under high-temperature conditions, combined with pathway analysis, the relative contributions of factors such as volatility and matrix-glycoside interactions to the transfer rate can be quantified.

### 5.4 The development of pyrolysis models and methods

During actual cigarette combustion, tobacco undergoes a non-isothermal, non-uniform thermal mechanism. In contrast, Py-GC-MS typically employs linear or instantaneous heating to a set temperature, lacking a realistic simulation of temperature gradients and heat transfer processes. The pyrolysis pathways and product distributions of glycosides vary significantly under different heating rates. Future research may employ multi-stage heating programs to simulate the temperature gradient of the combustion cone, combining with variable-atmosphere experiments, to more closely approximate the thermal mechanism and oxygen distribution during combustion.

### 5.5 Safety risk-based strategies for the use of glycosides

Existing Py-GC/MS studies indicate that glycosides may produce compounds posing potential health risks under pyrolysis conditions. The formation of harmful pyrolysis products exhibits temperature dependence. When most glycosides pyrolyze below 300 °C, the product composition remains relatively simple, with few harmful products present at low concentrations. Glycoside structure also significantly influences the safety of pyrolysis products. The following strategies can be adopted for safe glycoside application in cigarettes: (1) Prioritize glycoside types with pyrolysis temperature windows matching the cigarette combustion zone and lower harmful product formation. (2) Design glycosides with controllable pyrolysis pathways through chemical modification or biosynthetic methods, enabling complete pyrolysis at lower temperatures to avoid high-temperature secondary reactions.
